# Découverte fortuite d’un phéochromocytome en peropératoire

**DOI:** 10.11604/pamj.2018.29.142.13194

**Published:** 2018-03-05

**Authors:** Hicham Hachlaf, Abdelhamid Madani

**Affiliations:** 1 Service d’Anesthésie Réanimation, Centre Hospitalier Universitaire Mohamed VI, Oujda, Maroc

**Keywords:** phéochromocytome ectopique, paragangliome juxtapancréatique, pancréas, Ectopic pheochromocytoma, juxtapancreatic paraganglioma, pamcreas

## Abstract

Nous rapportons un cas inhabituel de phéochromocytome ectopique sans symptôme clinique. Les examens radiologiques montrent une tumeur développée au contact du pancréas. Durant l'acte opératoire après manipulation de la tumeur, une tachycardie, des pics hypertensifs et de troubles graves du rythme ventriculaire sont survenus, suivis d'une hypotension après extraction de la masse et installation d'un état de choc. La gestion des événements indésirables graves périopératoires révélant ce phéochromocytome méconnu est discutée.

## Abstract (en)

We here report an unusual case of ectopic pheochromocytoma without the presence of any clinical symptoms. Radiological examinations showed tumor in contact with the pancreas. Intraoperatively, after surgical manipulation of the tumor, the patient had tachycardia, hypertensive peaks and severe ventricular rhythm disorders followed by hypotension after tumor resection and shock. The management of severe intraoperative adverse events revealing pheochromocytoma is discussed in this case study.

## Introduction

Le phéochromocytome extrasurrénalien s'observe dans 10% des cas [1]. Sa découverte en per opératoire représente une entité rare et de très mauvais pronostic [2,3]. Le paragangliome est une tumeur chromaffine extrasurrénalienne issue du système neurovégétatif, c'est une tumeur rare pouvant se développer au contact du pancréas dont la ponction ou la résection chirurgicale peut entraîner des complications graves. Sa découverte peut être fortuite lors d'une intervention chirurgicale pour tumeur abdominale et être la cause de troubles du rythme parfois mortels. L'acte opératoire est associé à un risque majeur d'instabilité hémodynamique en l'absence d'une préparation et d'une surveillance anesthésique adéquate [2]. Nous rapportons le cas d'une patiente ayant présenté des pics hypertensifs majeurs avec instabilité hémodynamique suivi d'un état de choc au cours d'une chirurgie viscérale pour exérèse d'un paragangliome juxtapancréatique initialement pris pour une tumeur kystique pancréatique (cystadénome mucineux).

## Patient et observation

Mlle CS, âgée de 36 ans, sans antécédents pathologiques particuliers est hospitalisée en 2015 dans le service de chirurgie du centre d'oncologie pour une tuméfaction de l'hypochondre gauche apparue depuis 5 ans associée il y 'a un an à des douleurs épigastriques et vomissements intermittents. L'examen physique a retrouvé une sensibilité profonde du flanc gauche avec contact lombaire. Le reste de l'examen physique a été sans particularité, notamment la tension artérielle était à 130/70 mm Hg. Une échographie abdominale réalisée a retrouvé une volumineuse masse tissulaire de 11cm au niveau de l'hypochondre gauche et de la fosse lombaire gauche au contact du rein gauche sans point de départ précis. Une tomodensitométrie abdominale avec injection de produit de contraste réalisée a retrouvé une masse multiloculée au niveau de la queue du pancréas mesurant 10mm/8,6mm/10mm de diamètre, contenant de multiples lésions kystiques confluentes et dépassant pour la majorité 2 cm de diamètre, les septas et la paroi sont épaissies; la masse est responsable d'un refoulement du rein vers le bas sans l'envahir. Le diagnostic de tumeur kystique du pancréas et en premier lieu un cystadénome mucineux a été retenu, imposant un geste chirurgical. La patiente fut programmée pour une splénopancréatectomie caudale associée à une néphrectomie gauche. En consultation d'anesthésie, l'examen clinique retrouvait une hypertension artérielle modérée (PA = 150/80mmHg), une auscultation cardiopulmonaire sans particularité, une hyperglycémie à 1,47g/l et aucun critère prédictif d'intubation difficile. Le jour de l'intervention, la pression artérielle est 160/100mmHg et la fréquence cardiaque 93 bpm. Le monitorage per opératoire comporte un sphygmomanomètre de mesure automatique de la pression artérielle, un électrocardioscope et un oxymètre de pouls. La chirurgie a été réalisée sous anesthésie générale après une prémédication par 1 mg de midazolam, l'induction de l'anesthésie est réalisée avec 200mg de Propofol, 200μg de fentanyl et 0,6mg/kg de rocuronium, La patiente a été facilement intubée avec une sonde orotrachéale de 7mm et mise sous ventilation contrôlée. L'entretien de l'anesthésie générale associait du sevoflurane et des bolus de fentanyl et propofol. A l'induction, la patiente a présenté un collapsus tensionnel modéré, rapidement réversible après administration de 6 mg d'éphédrine.

L'incision chirurgicale a été pratiquée 15 minutes après l'induction anesthésique, par une voie d'abord sous-costale gauche; l'exploration a retrouvé une volumineuse masse refoulant le rein en bas et en arrière, au contact du pancréas et de la rate. Les conditions opératoires étaient bonnes. Vingt après l'incision, concomitamment à la manipulation de la tumeur, la patiente a présenté une hypertension artérielle progressive, malgré l'approfondissement de l'anesthésie et de l'analgésie puis un accès hypertensif à 280/155mmHg. Mais les chiffres tensionnels restent toujours élevés à 250/140mmHg, ce qui a motivé la mise en perfusion continue de nicardipine à la SAP à la vitesse de 4ml/h. Deux minutes après le début des accès hypertensifs, apparait une tachycardie supraventriculaire à 200bpm compliquée, les secondes suivantes, de tachycardie ventriculaire traitée par lidocaïne, à la dose de 1 mg/kg. Le diagnostic de phéochromocytome est évoqué à ce moment ce qui nous a incité rapidement à l'extraction de toute la masse. Après l'extraction, un collapsus tensionnel majeur est survenu (PA = 60/40mmHg) réfractaire au remplissage vasculaire et à l'administration d'éphédrine, qui a nécessité l'introduction de noradrénaline. Après ouverture de la pièce, l'aspect était évocateur d'un phéochromocytome par son aspect dense, brunâtre L'examen histologique de la tumeur confirme le diagnostic du phéochromocytome à localisation ectopique. L'étude anatomo-pathologique de la pièce opératoire a confirmé le diagnostic de phéochromocytome extrasurrénalien. Les suites opératoires sont simples et la patiente a été transférée en service d'endocrinologie. Dans les trois semaines suivantes, on a constaté une normalisation biologique des dérivés méthoxylés urinaires et plasmatiques. Sur le plan cardiovasculaire, l'état clinique et échocardiographique était sans particularité. Il a été convenu de réaliser une scintigraphie au méthyle iodo benzyl guanidine (MIBG) à distance.

## Discussion

Le phéochromocytome est une tumeur développée à partir des cellules chromaffines de la médullosurrénale ou d'autres ganglions sympathiques et secrétant en quantité variable des catécholamines [2]. Les symptômes révélateurs varient avec la substance produite et associent une hypertension (permanente ou paroxystique) et des accès de tachycardie, céphalées et sueurs. Lorsque le patient arrive en consultation d'anesthésie, le diagnostic est en général déjà fait et la prise en charge débutée, mais plusieurs cas cliniques de phéochromocytome méconnu découvert en pré-ou en per opératoire ont été décrits chez des patients opérés d'une autre pathologie et il faut savoir l'évoquer devant l'association d'une HTA et des accès paroxystiques de sueurs et de céphalées, récuser la chirurgie prévue et adresser le patient à l'endocrinologue [3, 4]. En per opératoire, l'apparition de crises hypertensives rebelles aggravées par les manipulations ou la c'lioscopie doit également alerter. La fréquence des phéochromocytomes est estimée a 0.5% des sujets hypertendus [4]. Le plus souvent, leur localisation est surrénalienne (80% des cas) et dans 20% des cas elle se situe le long de la chaine sympathique abdominale et thoracique [4]. Les paragangliomes sont des tumeurs chromaffines extra surrénaliennes issues du système neurovégétatif. Ils sont dits fonctionnels en cas de sécrétion de catécholamines. Développés aux dépens de cellules indifférenciées de la crête neurale primitive, ils se situent le long du squelette axial. Les principaux sièges sont le hile rénal (46%), l'organe de Zuckerkandl (29%) (glomus situé en avant de la bifurcation aortique près de l'origine de l'artère mésentérique inférieure), la vessie (10%), le médiastin postérieur (10%), la tête et le cou (2-4%) [5]. Bon nombre de phéochromocytomes restent méconnus et sont de découverte per opératoire, à l'occasion de troubles cardiovasculaires graves pouvant mettre en jeu le pronostic vital [6,7]. Plusieurs facteurs peuvent déclencher ces accidents: l'induction anesthésique, l'intubation trachéale, l'incision chirurgicale, la manipulation tumorale, enfin tout facteur pouvant stimuler la sécrétion des catécholamines [2]. Les complications cardiovasculaires survenant pendant la période opératoire sont dominées par les poussées hypertensives, les troubles du rythme et la défaillance cardiaque, surtout lors des accès hypertensifs. Le cauchemar de l'anesthésiste lors de sa découverte fortuite en per opératoire [8-12]. O'Riordan et al [13] rapportent une mortalité très élevée dans ce contexte, voisine des 80%. Malgré l'instabilité tensionelle de la patiente, nous n'avons pu renforcer la surveillance hémodynamique avec contrôle continu de la pression artérielle invasive et un accès veineux central, en raison de l'évolution très rapide du tableau clinique [14]. Dans notre cas, le contrôle des accès hypertensifs a reposé sur un inhibiteur calcique vasodilatateur, la nicardipine. Et en effet, les inhibiteurs calciques sont de plus en plus souvent utilisés pour traiter ces poussées d'hypertension artérielle peropératoire, d'autant que la survenue d'une hypotension artérielle entre les manipulations tumorales ou après exérèse, décrite avec tous les α-bloqueurs, ne semble pas observée avec les inhibiteurs calciques [15].

Avec ou sans préparation, il persiste toujours des risques d'instabilité tensionnelle au cours de la chirurgie du phéochromocytome. Dans ce cas, malgré la suspicion de phéochromocytome et le risque cardiovasculaire sous-jacent, la décision de terminer la chirurgie a été prise, car l'exérèse de la tumeur était presque finie et il ne restait plus qu'à réaliser la fermeture chirurgicale. Il est cependant conseillé, lorsque la chirurgie n'est pas trop avancée, de suspendre et reporter la chirurgie d'exérèse pour confirmer le diagnostic de phéochromocytome et permettre une préparation opératoire et une prise en charge anesthésique minutieuse [15]. Apres la première phase chirurgicale, marquée par les accès de libération paroxystique de catécholamines et l'intérêt des anti-hypertenseurs, survient un autre temps critique après l'exérèse tumorale ou au clampage veineux, dominé par le sevrage brutal en catécholamines. Concernant le cas de notre patiente, une fois la décision d'exérèse de la tumeur prise, un remplissage vasculaire massif a été entrepris pour anticiper le risque de collapsus tensionnel. Devant l'incertitude du diagnostic, qui a été évoqué rapidement, mais réfuté initialement par le chirurgien. Lorsque l'expansion volémique ne permet pas de normaliser la pression artérielle, le recours aux amines vasoactives, comme dans cette observation, peut être nécessaire pour corriger l'hypotension artérielle due à la chute brutale du taux des catécholamines et aggravée par une hypovolémie efficace préopératoire et la levée de la vasoconstriction chronique. Les suites de la chirurgie du phéochromocytome nécessitent une prise en charge initiale en soins intensifs ou en réanimation, en particulier lorsque l'état hémodynamique reste instable ou en cas de défaillance ventriculaire gauche. L'optimisation de la prise en charge du phéochromocytome en vue de son exérèse a permis une diminution significative de la mortalité périopératoire, passant de 25% il y a 60 ans à moins de 5% dans les années 1990 et pouvant même être nulle selon les séries les plus récentes [15, 16-18]. Cependant, les phéochromocytomes méconnus restent responsables de décès fortuits [19, 20], ce qui incite à renforcer l'effort diagnostique du vivant des patients. On peut, en somme, retirer de ce cas clinique l'importance de l'effort diagnostique d'une hypertension artérielle, d'autant qu'elle est associée à une tumeur abdominale. La prise en charge multidisciplinaire préopératoire doit favoriser la communication entre le chirurgien, l'anesthésiste réanimateur et le cardiologue pour échanger tous les éléments du cas clinique. De plus, il ne faut pas oublier d'évoquer le diagnostic de phéochromocytome devant une poussée d'hypertension artérielle brutale à l'induction anesthésique ou en peropératoire au cours des manipulations tumorales et ce après en avoir éliminé les autres causes. Enfin on peut retenir l'intérêt du contrôle tensionnel en préopératoire d'une chirurgie pour phéochromocytome par lesα bloquants ou les inhibiteurs calciques pour prévenir les effets de la sécrétion des catécholamines et diminuer ainsi la morbidité et la mortalité péri opératoire

## Conclusion

La fréquence élevée des phéochromocytomes méconnus et la multitude des diagnostics rapportés dans la littérature doivent attirer l'attention sur leurs manifestations graves pour les rattacher rapidement à leur cause. La chirurgie reste le seul moyen thérapeutique susceptible d'arrêter le processus lésionnel dû à l'imprégnation par les catécholamines. La gestion anesthésique des patients ayant un phéochromocytome est un challenge, particulièrement quand celui-ci n'a pas été diagnostiqué.

## Conflits d’intérêts

Les auteurs ne déclarent aucun conflit d'intérêts.
